# Video and App-Based Games for Youth Emotion Regulation: Scoping Review

**DOI:** 10.2196/96935

**Published:** 2026-09-09

**Authors:** Angela May Ashley, Jaidyn Kataryna Charlton, Hannah Wayne-Phillips, Aislin R Mushquash, Christine Wekerle

**Affiliations:** 1 Department of Psychology Faculty of Health and Behavioural Sciences Lakehead University Thunder Bay, ON Canada; 2 Department of Psychology Neuroscience and Behaviour McMaster University Hamilton, ON Canada; 3 Department of Pediatrics McMaster University Hamilton, ON Canada; 4 Optentia Research Unit North-West University Vanderbijlpark, North West South Africa

**Keywords:** youth, emotion regulation, video game, app, eHealth, serious games, digital intervention, digital health, scoping review, PRISMA, mobile phone, Preferred Reporting Items for Systematic Reviews and Meta-Analyses

## Abstract

**Background:**

Emotion regulation (ER) is a transdiagnostic construct encompassing distinct strategies that can be used to encourage adaptive coping skills in support of youth’s (ages 10 to 24 years) mental health. ER is responsive to training and intervention, such as via video and app-based games. Previous literature shows that video and app-based games impact the psychological well-being of youth and may be an accessible way to help youth learn about and implement emotional regulation strategies in their lives.

**Objective:**

Previous reviews have not focused on video and app-based games that support ER and how they may be beneficial to youth with and without a mental health diagnosis. This scoping review sought to collect, review, and understand the scope of research on the effects of video and app-based games on ER outcomes in youth, and areas in need of further investigation.

**Methods:**

We conducted a scoping search of MEDLINE, Embase, and APA PsycInfo from the beginning of the database up to and including December 2025. Studies were included if they were in English, included youth with a mean age between 10 and 24 years, and that evaluated an intervention or an intervention adjunct to help improve their ER skills using a video or app-based game. We extracted population characteristics (for the video game or app), the effectiveness of the intervention, and ER strategies addressed.

**Results:**

Following the screening of 14,456 studies, we identified 33 studies for inclusion in the final review. Four recurrent outcomes emerged: change in overall ER skills, mood modification, mental health symptom management, and emotion identification.

**Conclusions:**

While most studies reported improvements in these areas, some studies reported areas of continued difficulty. The results suggest that video and app-based games could serve as beneficial, easy-to-access tools to support youth ER through various strategies, with areas for future research to address mixed results and recommendations for addressing digital health improvement.

## Introduction

### Background

Adolescence is a prolonged developmental period, from ages 10 to 24 years [1]. However, terminology for this age range varies, often encompassing the terms “youth” and “young people” that are used interchangeably [2]. It is marked by neurocognitive variations in emotion regulation (ER [3]), including heightened emotional volatility [3], differences in self-concept [4], increased risk-taking behavior [5], and a greater emphasis on reward attainment [6]. Despite these changes, youth can modulate and change how they respond in stressful circumstances using key strategies to regulate and manage their emotional responses, formally known as the process model of ER [7].

### ER

ER is defined as “the management and organization of diverse systems and components, including internal systems (ie, neurophysiological, cognitive, and subjective evaluations), behavioral components (ie, facial and behavioral actions), and external/social components (ie, cultural values, social contextual significance, personal motivation/goals)” [8]. ER is also identified as a transdiagnostic treatment target for youth to facilitate adaptive emotionality and coping [9]. According to the process model of ER by Gross [7], ER is composed of 5 distinct strategies, including situation selection, situation modification, attentional deployment, cognitive change, and response modulation [10]. Any number and combination of these strategies may support youths’ response modulation to stress [11]. From an intervention perspective, attentional deployment, cognitive change, and response modulation are amenable to effort. For instance, individuals may use techniques such as deep breathing to modulate their physiological arousal and reduce the likelihood of stress reactivity [7,10,12,13]. Tracking personal mood influences the deployment of attention (toward a more positive mood) and may promote cognitive change in how negative emotions are perceived and appraised. As such, short-term goals may be motivated hedonically to feel good or better, which can contribute to long-term goals such as problem-focused coping [13,14]. This process model provides a cycle of valuation and regulation that youth can use to guide their everyday interactions and experiences. Therefore, through appropriate ER, youth can effectively manage and reduce negative emotions, as well as enhance and maintain positive emotions, thereby better meeting their needs or goals [15].

### Video and App-Based Games and ER Among Youth

The majority of youth have access to digital devices, including smartphones (95%), gaming consoles (80%), and desktop or laptop computers (90%) [16]. Through these devices, youth are the most likely to engage with video games (73%) in a typical week and, among those who have their own smartphone (96%), most (58%) download “apps” on their mobile devices [17,18]. According to a 2023 US Pew Research Center survey, 85% of US youth play video games, with 41% of youth playing daily, and 4 in 10 youth identifying as “gamers” [19]. Of those who endorse playing video games, significant gender gaps exist, with 97% of boys endorsing playing games, compared to 73% among girls [19]. Despite this, youth in general are using digital devices and playing digital games at a rapidly growing rate.

There has been a growth in the use of digital health tools (eHealth) to support youth mental health and well-being [20]. In a review of digital interventions, most studies (n*=*27; 69%) included digital games, and reported a small but significant impact on reducing negative emotional experiences, especially anxiety in youth at risk of developing a mental health disorder [21]. However, despite digital interventions improving ER, this overall effect did not achieve significance. As such, it remains relevant to isolate the impact of digital games on ER.

In their review, Reynard et al [21] defined “digital games” as electronic games that “function to achieve specific goals,” with many of their included studies containing video or app-based games (ie, mobile apps containing or functioning as games). However, video and app-based games in the mental health literature have commonly fallen into the overarching categories of “casual” and “serious” games. While casual games aim to be a source of entertainment (often arcade-like games that are fun and easy to learn), serious games involve the use of games or game elements to provide psychoeducation and skill-building (eg, stress reappraisal), with a specific goal, such as improving attitude or achieving behavior change [22,23]. While both casual and serious video and app-based games can contain a variety of game features (eg, goals, levels, and narratives), specific aspects of gaming, such as control and mastery features, immersive and customizable experiences, and feedback and reward mechanisms, have been identified as potential contributors to increased ER through physiological and emotional self-regulation [14,24]. This aligns with the cultivation of the attentional deployment, cognitive change, and response modulation facets of the process model by Gross [7].

Other subcategories of video and app-based games also appear as part of casual and serious games, addressing components of this model. Notably, common game types may include biofeedback, adventure, puzzle, and challenge games. Biofeedback games consist of using the participants’ biometrics, such as heart rate, respiration, or neurofeedback, to control some aspect of the game [25]. Adventure games involve those in which participants interact with characters and follow a story, often involving a fantasy space for players to obtain knowledge and understanding as they progress the narrative [26]. Puzzle games include games that require putting objectives or pieces together, while flexibly adapting to changing rules as progress is made [27]. Lastly, challenge games include those that involve working toward skill-testing but achievable tasks, and may include more explicit reward systems (eg, points and skill attainment) [28]. Despite the scope of video and app-based games, there remains a need to synthesize how such characteristics of video and app-based games help target ER among youth.

### Study Objectives

To date, despite the role that various video and app-based games appear to play in supporting mental health and emotional well-being among youth, no studies have reviewed the transdiagnostic construct of ER and related strategies in such games as interventional targets among youth. This scoping review explored the existing research on the impact of video and app-based games on ER in youth to identify knowledge gaps and the current landscape of ER findings. We applied the TEME (target audience, engagement, mechanisms of action, and effectiveness) framework [29] that highlights key themes in the development of game-based digital mental health interventions: target audience (T), levels of engagement (E1), mechanisms of action (M), and health-related effectiveness (E2).

Across the studies in this review, the T for gaming was youth with a mean age between 10 and 24 years, consistent with the literature reporting high video game and app usage among this age range [17,18]. This also accounts for the developing brain networks supporting ER in this age group (ie, prefrontal cortex involvement in emotional control, response modulation, and valence learning), and both executive functioning and social processing that advance from youth into the young adult years (ie, working memory, perspective-taking, and decision-making [3]). Corresponding identity variables across the included studies (eg, ages, gender, and mental health status) are reported. E1 examines whether studies reported the degree of interaction with the gaming intervention (eg, duration and frequency of use) and degree of immersion, conceptualized here as game modality (eg, virtual reality [VR] and computer-based). While user motivation to use the gaming intervention is also a metric of engagement, this is beyond the scope of this review [29]. We also consider the M, that is, the routes through which a digital intervention achieves behavioral and symptom-level changes, which are captured through the video and app-based descriptions (eg, modeling behavior, self-reflection, and skill development) [29]. Lastly, we considered the effectiveness (E2) of video and app-based games in achieving behavioral and symptom-level changes among participants in the studies. This included, but was not limited to, changes in a youth user’s capabilities, motivation, or training of new outlooks and behaviors that can ultimately result in changes in their symptoms or presenting concerns [29]. Overall, this scoping review addresses the following research question: what is the scope of game-based ER interventions, particularly in terms of video and mobile app-based games, and their corresponding ER outcomes among youth?

## Methods

### Study Design

We conducted a scoping review as a preliminary evaluation of this topic area to determine the breadth of available research and clarify conceptual boundaries [30]. This review followed the PRISMA-ScR (Preferred Reporting Items for Systematic Reviews and Meta-Analyses extension for Scoping Reviews) checklist (Multimedia Appendix 1), adhering to systematic procedures to collect and analyze data to maintain transparency and the possibility of replication [31].

### Search Parameters and Study Selection

We searched 3 electronic bibliographic databases: MEDLINE, Embase, and APA PsycInfo. A search strategy was developed in consultation with an academic librarian. The search terms included the major concepts of “video games,” “apps,” and “emotional regulation” (Multimedia Appendix 2). Our search expanded with the use of database-specific index terms and related keywords as necessary; however, we generally sought to keep the searches quite broad and all-encompassing in order to retrieve as many potential studies as possible. For all databases, we searched from the beginning of the database up to and including December 2025. Papers were uploaded to Covidence and Rayyan to facilitate title and abstract review to determine citation relevance. Both platforms were used due to an institutional shift in platform access, but show reasonably comparable utility in supporting blinded screening, while the authors took steps to document procedures as described to uphold the integrity of the review process [32]. Three reviewers removed duplicate papers before screening. Three reviewers independently screened all titles and abstracts to determine relevance. Screening of the full texts was then completed independently by 2 reviewers. Disagreements during the screening of titles, abstracts, and full texts were resolved by a third independent reviewer or through a discussion among all the reviewers until a consensus was reached.

### Inclusion Criteria

The researchers considered studies for inclusion if the study evaluated an intervention or an intervention adjunct to help youth (between the ages of 10 and 24 years) improve their ER skills by using a video game or an app that has games in it. To understand the types of games being used as ER interventions among youth, we included both casual and serious games to consider their relative impact on ER outcomes. Given that video and app-based games can contain many types of features (eg, feedback and reward) and can be administered through multiple modalities (eg, mobile devices, computers, and VR platforms), these criteria were not restricted in order to maximize the scope of our findings and limit inadvertent omission of relevant data. As such, at a minimum, a “video game” or “app-based game” was defined in alignment with Reynard et al [21] as electronic games (regardless of modality) that function via user interaction to achieve a goal or outcome. Psychological, psychiatric, or emotional indicators as outcomes were included (eg, anxiety, depression, and ER). Additional inclusion criteria included studies that examined primary data, were available in English, were published in a peer-reviewed journal, and were available as a full text.

The reviewers excluded reviews, commentaries, editorials, book chapters, dissertations, theses, protocols, abstracts, or papers not using primary data. Excluded studies included those with multiple interventions whose effects could not be disentangled from the use of video games independently. We excluded studies that did not specify the mean age of their participants or included age bands whereby the youth demographic’s findings could not be independently assessed from adult age bands defined within the studies. Excluded studies included those that did not implement the use of a video game or an app with games as an intervention. We selected this criterion to acknowledge that the use of video games in a noninterventional setting may be related to different outcomes (eg, to provide entertainment [33]) compared to an interventional setting focused on ER and goal-directed outcomes. The research team excluded studies focused on video game addiction, internet gaming disorder, or violence and aggression associated with video games. Notably, addictive and problematic gaming could interfere with interpreting the effects of video and app-based game interventions (eg, variation in reward circuits and compulsive tendencies among problematic gamers [34]), with studies evaluating violence and aggression focusing on links between aggression and video games, rather than video games as interventions for aggression [35]. Excluded studies included physical games or “exergaming” studies, as the effects of these video games vs engagement in physical activity on the outcomes could not be delineated. We excluded studies that evaluated the use of video games perioperatively or during uncertain medical situations (eg, chemotherapy and pain management). This criterion was necessary because emotional changes induced in these settings are distinct and beyond the scope of this review [36].

### Data Extraction and Analysis

Two reviewers independently extracted detailed study characteristics. The same reviewers also independently coded the papers. One reviewer assessed the extractions and coding schemes for consensus. A descriptive (eg, study characteristics) and narrative (eg, “data stories,” key recurring patterns, or emerging themes) analysis was conducted [37]. We indexed included studies in our review based on the above eligibility criteria and 2 reviewers charted the following information (Multimedia Appendix 3 [38-70]) that was jointly developed and approved by the research team for consistency: authors, title, DOI, year of publication, sex and/or gender (whichever was available) of the participants, population descriptions (eg, location if available), mean age and range of participants, inclusion criteria, exclusion criteria, sample size, name and description of intervention and video game, type of game (eg, serious or casual), subcategory of game (eg, biofeedback, adventure, puzzle, and challenge), game modality (eg, computer-based and mobile device), primary findings, study limitations, and main ER outcomes.

In alignment with the TEME framework [29], study characteristics listed above are mapped onto each domain in Multimedia Appendix 3, including the T included in the study (ie, youth with or without a diagnosed mental health condition), the intervention description (ie, how the game was engaged with, including modality), the game description (ie, the game’s M as an intervention), and the effectiveness (eg, study design, main outcomes, and author-identified limitations). Summaries reflected the authors’ descriptions. When the authors did not describe a game, efforts were made to identify the information through an internet search (eg, type of game or game website) or the authors explicitly stated that details were not provided.

## Results

### Selection and Inclusion of Studies

**Figure 1:**
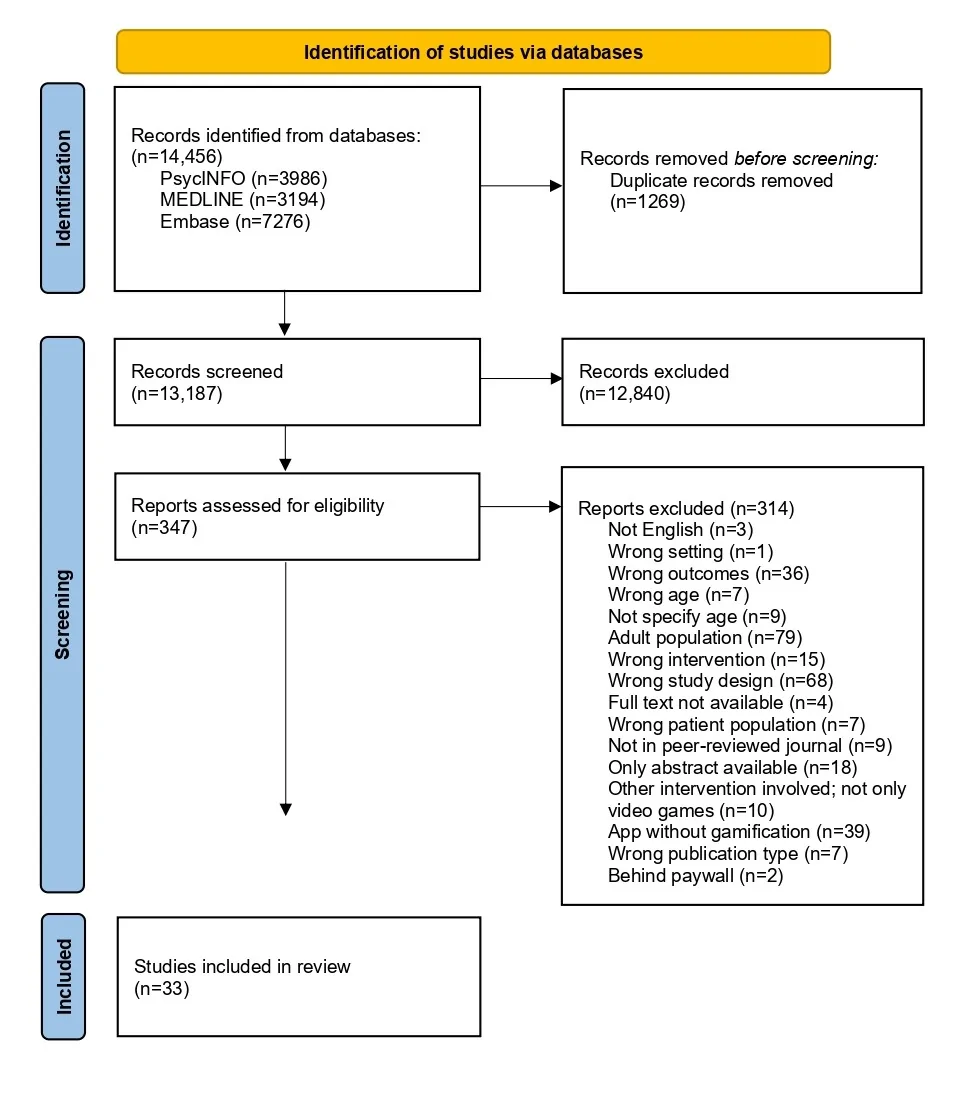
PRISMA (Preferred Reporting Items for Systematic Reviews and Meta-Analyses) flowchart.

We identified 14,456 citations from our search of 3 electronic databases. After the removal of duplicates, we screened 8597 titles, 4590 abstracts, and 347 full-text citations. After applying the exclusion and inclusion criteria, 13,154 studies were removed, resulting in the final review of 33 studies (Multimedia Appendix 3), as illustrated in the PRISMA (Preferred Reporting Items for Systematic Reviews and Meta-Analyses) diagram (Figure 1). The following sections outline the primary results of these studies in alignment with the TEME framework (Table 1) [29].

**Table 1:** Main TEME^a^ framework findings across studies^b^.

TEME framework	Main findings
Target audience	Studies mainly consisted of nonclinical (n=23) midadolescent youth (mean age 16.04, SD 3.63 years). The majority of samples included male and female participants. Sample sizes varied across studies, ranging from an average of 25.25 (qualitative, SD 16.15) to 237.12 (quantitative, SD 387.88) youth. Additionally, study designs primarily consisted of randomized controlled trials (n=12) or observational studies (n=5).
Levels of engagement	Across studies, on average, the prescribed video game or app engagement included 6 weeks of total use (SD 4.89), including an average of 12.27 sessions (SD 13.74). Prescribed gameplay averaged 40.28 minutes (SD 21.57). Most studies used computers or laptops to host the games.
Mechanisms of action	We identified 28 different video and app-based games across the included studies. Game types mainly consisted of serious games (n=26), with adventure (n=11) and challenge (n=7) games appearing most often as subcategories and associated with positive ER^c^ outcomes. Across game descriptions, primary mechanisms of action included ER skills, including psychoeducation, resilience-building, coping strategies, cognitive reframing, decision-making, problem-solving, relaxation, and self-monitoring. The majority of games were designed to be played individually.
Effectiveness	Overall, studies primarily reported positive (n=16) and mixed (n*=*15) findings following video and app-based game use, with 2 studies reporting null results. ER outcome domains included change in overall ER skills (n=16; eg, reported increases in coping skills and self-expression), mood modification (n*=*12; eg, reported increases and decreases in positive and negative affect), mental health symptom management (n=11; eg, mixed anxiety outcomes and reported improvement in prosocial behavior), and emotion identification (n=4; eg, reported improvement in emotional awareness). However, key caveats include many of the studies reporting limitations such as a small sample size and being underpowered (n=19), participant homogeneity (n=5), and needing longer-term follow-up (n*=*9).

^a^TEME: target audience, engagement, mechanisms of action, and effectiveness.

^b^More details can be found in Multimedia Appendix 3.

^c^ER: emotion regulation.

### Characteristics of Included Studies and T

Multimedia Appendix 3 provides a consolidated overview of the included studies. Studies were from 2009 to 2025, and across multiple countries: the Netherlands (9), United States (6), Romania (5), Canada (4), Spain (3), United Kingdom (2), China (2), Australia (1), and Switzerland (1). Studies were quantitative (n*=*29), qualitative (n=2), and mixed (n=2) in design. The majority of studies consisted of randomized trials or randomized controlled trials (RCTs, n=16; see Multimedia Appendix 3 for additional study design parameters).

Regarding the T, the average sample size for quantitative studies (including the mixed quantitative participants) was 237.12 (SD 387.88) participants, with a range from 8 to 1871 youth. The average sample size for qualitative studies (including the mixed qualitative participants) was 25.25 (SD 16.15) participants, with a range from 6 to 30 youth. Studies mainly focused on midadolescent youth, with quantitative study mean ages ranging from 10.03 to 24.00 years, and qualitative study mean ages ranging from 14.24 to 18.77 years. The average age for the quantitative studies is 16 (SD 3.70) years, and for the qualitative studies is 15.76 (SD 1.79) years. Twenty-four studies included both male and female participants, 3 studies included both men or boys and women or girls, 5 studies included both the sex or gender categories above with another gender diverse identity (or were otherwise unspecified), and 1 study included only female participants. In terms of presenting concerns, 23 studies included nonclinical youth who did not have any diagnosed mental, psychiatric, or behavioral concerns (n=17) or presented with subthreshold symptoms (n=6). Of the 10 studies that focused on clinical youth (note that some studies addressed multiple diagnoses), foci included a combination of symptoms and/or specified and unspecified diagnoses (n*=*5), anxiety (n*=*1), anger or externalizing symptoms (n*=*1), posttraumatic stress disorder (PTSD, n*=*1), autism spectrum disorder (ASD, n*=*1), or attention-deficit/hyperactivity disorder (ADHD, n=1).

### Engagement (E1) in Video and App-Based Games

When considering E1, duration of video and app-based game interventions ranged from 1 week to 6 months, with an average of 6 (SD 4.89) weeks of prescribed engagement (among studies that reported durations of game engagement). Studies reported that the prescribed dosage of game use ranged from 1 to 42 sessions, with an average of 12.27 (SD 13.74) sessions (among studies that reported prescribed session counts). Studies reported an average of 40.28 (SD 21.57) minutes of prescribed gameplay, with studies ranging from 10 to 90 minutes. Five games did not have any prescribed use parameters (SquareMoves, CharacterMe, Reach Out Central, SmartCAT 2.0, and EmoWELL). Only a small number of studies reported usage metrics following their game intervention [38-42] (Multimedia Appendix 3). Regarding levels of immersion, computers (eg, desktop and laptop) hosted most games (15). Alternative platforms included mobile devices such as phones and tablets (9), VR devices (2), or were multiplatform (ie, VR or computer, 2).

### Game Types and M

In total, we identified 28 different video and app-based games across the 33 studies. Of these, we identified 6 games examined across multiple studies (REThink [43-47], JoyPop/SquareMoves [38,39,48,49], Mindlight [50,51], Deep [40,52], Dojo [53,54], and Grow It! [55,56]), and 22 games examined in 1 study (empowerED [41], SmartCAT 2.0 [42], Reach Out Central [57], RAGE-Control [58], Bejeweled 2 [59], Bookworm Adventures [59], Peggle [59], Muse [60], DayDream [60], Wild Divine [60], Grow Your Chi [61], Free Rice [61], Snake [61], Star Conflict [62], Happy Sport [63], music-based Roblox Game [64], EmoWELL [65], The Secret Trail of Moon [66], The Climb 2 [67], ReGoal [68], CharacterMe [69], and iTRAC [70]). Three studies examined multiple games in one [59-61]. Twenty-six studies investigated serious games, and 7 studies investigated casual games. Among the serious and casual games, 4 subcategories of games emerged, with some falling into more than one category. Of these, 11 studies investigated adventure games, 7 studies investigated challenge games, 5 studies investigated biofeedback games, 5 studies investigated puzzle games, 3 studies investigated biofeedback or adventure games, and 2 studies investigated puzzle or challenge games. Video and app-based games also achieved their corresponding ER outcomes through ER skill practice, including psychoeducation, resilience-building, coping strategy use, cognitive reframing, decision-making, problem-solving, relaxation, and self-monitoring. The majority of games appeared to be administered individually (n*=*25), with some administered in a group setting (n=3).

### Main ER Outcomes and Effectiveness (E2) for Youth Well-Being

Overall, the reported ER outcomes across the included studies were primarily positive (n=16), followed by 15 studies reporting mixed (eg, positive with null or negative outcomes) and 2 studies reporting null outcomes (ie, no significant effect). The majority of positive (n*=*13) and mixed (n*=*11) outcome studies involved serious games, with both null studies involving serious games. Additionally, the majority of video and app-based games with positive outcomes were in the subcategories of adventure (n=8) and challenge (n=5) games.

Across the included studies, 4 recurrent ER outcomes emerged as analytic groupings in this scoping review as a result of using video and app-based games and reflect distinct ER-related constructs. The first outcome noted the change in overall ER skills, which included studies that used tools that explicitly measured ER facets, including resilience. The second outcome was mood modification*,* which included study outcomes that focused on mood and affective change (ie, one’s emotional state or feeling). The third outcome was mental health symptom management, which included studies that aimed to manage symptoms (eg, improve psychological or behavioral well-being) among those with and without mental health disorders, representing broader outcomes of skills application (eg, cognitive change and response modulation) [7,10]. The fourth outcome was emotion identification, which included studies that aimed to improve the perception, identification, and understanding of emotions and conceptually serves as a precursor to ER skill use [71].

### Change in Overall ER Skills

Sixteen studies measured ER skills and changes in ER and corresponding secondary outcomes. Across randomized trials and RCTs [41,44,47,64,66,70], the studies identified improvements in overall ER skills, alongside secondary outcomes as a result of this. Specifically, David et al [44] used the emotion-regulation index for children and adolescents to measure ER, including the emotional control and emotional self-awareness subscales, and reported improvements in ER following the use of the REThink mobile video game. Fernandes et al [41] reported that after playing empowerED, there was an increase in cognitive reappraisal and positive beliefs that were associated with an increase in youth positive self-perceptions. Houck et al [70] reported that, following the use of iTRAC, youth self-reported improvements in their ER competency and greater use of ER strategies. Li et al [64] suggested that increases in self-efficacy lead to increases in ER and positive affect in association with the use of an unnamed music app. David and Fodor [47] reported significant improvements in ER, mental health difficulties, and more adaptive coping among youth who experienced maltreatment following the use of the REThink mobile video game. Alternatively, a study by Martin-Moratinos et al [66], who used the Strengths and Difficulties Questionnaire and included an ER subscale, suggested that playing The Secret Trail of Moon did not significantly improve ER in clinical youth with ADHD. However, they observed significant improvements in material organization, working memory, and inhibition.

Across nonrandomized studies [38,39,42,48,49,57,63,65,68,69], results suggested that youth demonstrated increases in ER, including the use of particular regulation strategies (eg, problem-based coping and seeking social support) among youth with higher levels of self-reported adverse childhood experiences (JoyPop [38]), and those experiencing stressful sporting situations (Happy Sport [63]). Notably, Ros-Morente et al [63] reported that, in the context of a sports game (Happy Sport), learning ER strategies helped youth feel increased sports satisfaction and was associated with reductions in bullying perpetration. Charlton et al [48] also reported that using JoyPop was associated with increased ER in a clinical sample of Indigenous youth receiving mental health support. Malik et al [49] further reported that clinical youth JoyPop users self-reported increases in self-awareness and self-expression of their emotions and thoughts. Livanou et al [68] suggested in their study that practicing prosocial skills while using ReGoal helped youth with conduct disorder regulate their emotions and was associated with reduced impulsivity. After using EmoWELL, Velert-Jimenez et al [65] reported significant improvements in ER and associated reductions in expressive suppression, emotional rejection, and overall emotion dysregulation. Similarly, Silk et al [42] suggested that using SmartCAT 2.0 among clinical youth receiving cognitive behavioral therapy treatment resulted in improved thought challenging and reduced avoidance of fears post treatment.

However, studies suggested that there was limited effectiveness among participants with preexisting anxiety and depression. Shandley et al [57] identified that despite there being increases in the use of problem-based coping after playing Reach Out Central, male youth experienced an associated nonsignificant decrease in resilience (though both genders demonstrated higher mental health literacy and help seeking). Qualitative studies incorporating youth and parent interviews assessed perspectives on ER abilities after game use [39,49,69] and reported mixed findings. While these studies reported increases in emotion coping, expression, and management following gameplay (SquareMoves in JoyPop), others suggested no significant changes in associated ER over time (CharacterMe [69]).

### Mood Modification

After playing video games, 12 studies identified pre- and postgame mood modification, including David et al [44-46], Dietvorst et al [55], Li et al [64], Livanou et al [68], MacIsaac et al [38], Mens et al [56], Mushquash et al [39], Russoniello et al [59], Tamplin-Wilson et al [61], and Weber et al [62]. This included results that suggested participants demonstrated decreases in depressive mood (REThink [44]), in irrational beliefs leading to decreases in negative and depressive mood scores (REThink) in a nonclinical [45] and clinical [46] sample, as well as associated increases in positive emotions, affect, and cognitive well-being (music-based Roblox game [64]). Study results suggested that this was especially promising among youth with higher risk profiles, including those with more depressive symptoms [55] and a higher number of adverse childhood experiences [38]. Mushquash et al [39] also reported that the JoyPop app supported mood check-in to facilitate taking steps for improvement among youth users. Russoniello et al [59] reported that, following 20 minutes of gameplay with Bejeweled 2, Bookworm Adventures, and Peggle, youth demonstrated improvements in mood alongside reductions in depression and tension.

Following the use of a negative mood-induction computer game, Cyberball [72], where participants were either passed a virtual ball or excluded to induce feelings of ostracism, video game playing through Grow Your Chi also suggested an associated increase in positive affect scores and a decrease in negative affect scores [61]. Notably, Tamplin-Wilson et al [61] reported that mood repair can be achieved through repairing relational needs via video games, including through improved self-esteem and belonging (ie, the game Grow Your Chi) as well as related improvements in prosocial behavior (ie, the game Free Rice). Additionally, Livanou et al [68] reported that using the mobile app game ReGoal to improve prosocial skills was associated with reduced negative affect and enhanced mood among youth with conduct disorder.

Alternatively, 1 study, Mens et al [56], reported that, following the use of the Grow It! app, youth, on average, exhibited decreased positive affect and adaptive coping with an increase in negative affect. However, among those with fewer depressive and anxiety symptoms, results suggested that Grow It! increased their positive affect while reducing negative affect. Similarly, Weber et al [62] reported that youth demonstrated improved mood repair and lowered stress levels after playing Star Conflict. However, results suggested that participants’ high and medium levels of presence (ie, being immersed in the game) significantly predicted their mood repair for positive emotions.

### Mental Health Symptom Management

Regarding mental health symptom management, 11 studies involving nonclinical samples primarily addressed changes in stress and anxiety, including Dietvorst et al [55], Li et al [64], Livanou et al [68], Mushquash et al [39], Russoniello et al [59], Scholten et al [53], Shandley et al [57], Weber et al [62], Weerdmeester et al [52], Wols et al [51], and Zhang et al [67]. Across these studies, results suggested that video and app-based games were associated with improvements in anxiety symptoms among youth with subthreshold depressive symptoms (unnamed music app; Li et al [64]), general anxiety (Grow It! [55]) with no differences by sex or age groups (Dojo [53]), lowered stress (Star Conflict [62]), and reductions in tension (Bejeweled 2 [PopCap Games], Bookworm Adventures [PopCap Games], and Peggle [59]). Zhang et al [67] used the Symptom Checklist-90 to measure overall mental health, which included subscales to assess fear and anxiety, and results suggested associated improvements in overall mental health and significant reductions in anxiety and fear symptoms following the use of The Climb 2 (Crytek). Similarly, the youth interviews by Livanou et al [68] revealed that using ReGoal was associated with reductions in their anxiety levels and was reported to have improved their relaxation among those with conduct problems.

In contrast, Weerdmeester et al [52] reported that there was no significant difference in associated decreases in state anxiety symptoms in a neurofeedback video game (Deep) compared to a normal guided breathing activity. Interestingly, Wols et al [51] investigated how expectations of the same video game (MindLight) may influence how such games have an effect based on whether youth are aware or not of the video game’s therapeutic potential. However, results suggested that such therapeutic expectations did not contribute to any differences in the reduction of state anxiety for youth and, on the contrary, showed related increases in state anxiety. Additionally, Shandley et al [57] examined how a video game (Reach Out Central) helped provide strategies to youth to support coping with psychological distress. Notably, the study reported improved resilience scores among female participants, while male participants exhibited decreased resilience scores, though this finding was nonsignificant. One study by Mushquash et al [39] also reported that the JoyPop app led to self-reported increases in stress among youth due to app functionality (eg, too many buttons and colors). However, this finding arose among a small subset of the participants.

We also identified 9 studies addressing clinically impairing symptoms among those with a mental health disorder, including anxiety [40,42,54], ASD [50], aggression and oppositional behaviors [58], conduct disorder [68], ADHD [66], and PTSD symptoms [60]. An additional study included clinical participants with undisclosed mental health concerns [49]. Anxiety-related outcomes included associated reductions in overall symptoms (DEEP and Dojo, though improvements did not last post treatment [40,54]) and no longer meeting diagnostic criteria (SmartCAT 2.0 [42]). Qualitatively, youth interviewed by Malik et al [49] stated that the SquareMoves game and the JoyPop app offered a distraction during times of stress and anxiety, and also reported feelings of reduced stress with use. However, while Wijnhoven et al [50] examined youth with ASD and subclinical anxiety symptoms, they reported that, despite girl participants and parents reporting significant decreases in anxiety symptoms after the video game intervention (MindLight), the overall decrease in child-rated anxiety was not significant compared to the control group. Whether these anxiety symptoms were directly related to the youth’s diagnosis of ASD was unknown. Additionally, Martin-Moratinos et al [66] used the Swanson, Nolan, and Pelham Rating Scale and the Conners Abbreviated Symptom Questionnaire to measure parent-rated changes in ADHD symptoms following the use of The Secret Trail of Moon, but did not report any significant contributing differences to main ADHD symptoms. However, they noted significant improvement in material organization, working memory, and inhibition, with clinical trends of improvement (values approaching significance) in main ADHD symptoms among the more engaged youth using The Secret Trail of Moon. One study also reported significant decreases following gameplay in overt, clinically impairing aggression and oppositional behaviors alongside decreases in median heart rate as a reflection of enhanced self-regulatory capacity (RAGE-Control [58]). Following the use of ReGoal, Livanou et al [68] reported that youth with conduct disorder self-reported related increases in empathy, prosocial behavior, and improved interpersonal relationships. Lastly, study results suggested that youth with clinical levels of PTSD symptoms experienced associated improvements in their symptoms, as well as improvements in depression and aggression, with the use of the Muse videogame, while the other 2 video games included in the study (DayDream and Wild Divine) showed inconsistent improvement associated with gameplay [60].

### Emotion Identification

Four studies reported video and app-based game outcomes contributing to emotional awareness and understanding. In 1 study, David et al [43] used Feeling Better (part of the REThink therapeutic online platform) with the goal of teaching and testing youths’ ability to recognize and understand the difference between functional and dysfunctional emotions. Notably, after just 3 gameplay trials, the findings supported these outcomes. Interview findings from the study by Mushquash et al [39] suggested that youth participants experienced improvements in their ability to identify their emotions and reported related increases in their general awareness of their emotions associated with the use of the JoyPop app, including the use of the app-based game, SquareMoves. Similarly, the study by Silk et al [42] reported that youth displayed improved emotion identification following the use of SmartCAT 2.0. Lastly, in a study from Velert-Jimenez et al [65], youth reported that using EmoWELL was associated with improvements in their introspection, awareness, and identification of emotions.

### Study-Reported Limitations

The studies included in this review represent a heterogeneous mix of designs and methodologies for assessing ER outcomes among youth, with the quality of evidence varying across studies. The majority of studies (n*=*19) indicated a “small” sample size as a primary limitation, with many endorsing a small effect size and being underpowered due to this. Nine studies endorsed issues with participant homogeneity (eg, mostly female participants or girls), lacking a control group, and measure limits (eg, self-report and not intended for the population being studied). Eight studies noted that they did not track changes in desired outcomes over a longer period of time, limiting their investigation of longer-term intervention effects. Seven studies reported attrition or compliance issues (eg, lack of continued use). Similarly, 5 studies reported a lack of environmental control (eg, exposure to other similar programs and unstructured use) during the study. Lastly, some studies reported issues with not including usage metrics (eg, duration and frequency of use, n*=*2), a lack of randomization to study conditions (n*=*1), and no blinding of experimenters in the study phases (n*=*1).

## Discussion

### Principal Findings

This scoping review sought to examine the breadth of research on game-based ER interventions, specifically when considering the impact of video and app-based games on ER outcomes among youth.

Given the centrality of ER skills underlying many mental health concerns and treatment approaches, it is essential to examine how video and app-based games may impact youth wellness outcomes as accessible ER support tools that are already at their disposal. In particular, the review identified key TEME findings alongside 4 ER outcomes that offer valuable insight into the current ER strategies being supported through the use of video and app-based games for youth (eg, attentional deployment and response modulation [7]). Namely, these included changes in overall ER skills, mood modification, mental health symptom management, and emotion identification.

### TEME Findings

When considering the T of the studies included in this review, they predominantly included nonclinical youth in the midadolescent age range (approximately 12 to 15 years) to young adulthood (approximately 24 years). While the definition of “youth” varies across the literature, this was in alignment with the target youth population and age ranges anticipated to be most likely to engage with video and app-based games in this review.

Among the included studies, E1 varied greatly across studies, with limited integration of usage metrics to assess actual vs prescribed use of the video and app-based game interventions. Regarding immersion, many of the games were administered through a computer or mobile device. This aligned with the M of most games, with positive ER outcomes being adventure- or challenge-type games. As such, despite most of the serious games across the studies incorporating biofeedback components, this level of immersion does not appear to be necessary for youth using these video and app-based games to experience positive outcomes. Alternatively, adventure and challenge components seem to be aligned with more positive outcomes, even among the casual games included in the review. Additionally, all games included some degree of ER skill development, with many focused on psychoeducation, resilience-building, and relaxation.

Lastly, when considering (E2) across video and app-based games, results appeared to split between positive and mixed outcomes for both casual and serious games. Given the positive outcomes exhibited by both game types, these outcomes may suggest the benefit of considering app development frameworks that incorporate both serious and casual gamification features (ie, “activist-casual game design,” see the study by King [24]) to address the shortcomings of each with improved study design. The following sections provide additional considerations in alignment with each of the identified ER outcomes.

### Change in Overall ER Skills

Overall, 16 studies reported improvement in ER skills in this review. These studies included those that benefited from the robust nature of an RCT design, used established ER measures, incorporated a longitudinal design, or were qualitative studies assessing changes in ER after gaming engagement to support a mixed-method research base for games and app use. In particular, many of the aforementioned games and apps used a virtual avatar or roleplay that implemented modeling and interactivity from the user to promote such ER skill development. This included skills such as cognitive reappraisal, emotional self-awareness, control, expression, and adaptive coping. In much the same way that youth may benefit from emotion modeling through a care provider or clinician, video and app-based games may offer another avenue for emotion modeling through virtual avatars and role-playing games that encourage the application of such skills in daily life, encouraging generalizability to their own interactions [73]. These actions also relate back to the process model of ER, including situation modification and selection, in which users may be empowered to take the necessary steps to modify their external environment and engage in positive social interactions that, in turn, improve their ER [7]. However, future research may wish to consider under what conditions this modeling with avatars and roleplay is most effective, given the limited literature on this area [73]. Additionally, as technology evolves and may integrate newer AI components into such game features, it will be important to consider how sustained video and app-based game engagement may lead to multidimensional fatigue and strain from AI-related interaction and decision-making [74].

Secondary findings suggested that experiencing self-efficacy provided by these games is associated with improved ER. With these findings in mind, providing opportunities for success through in-game challenges or winnings may be targeting youth’s reward system response [14], which, in turn, may help improve their feelings of self-confidence and success in implementing new emotional coping skills. Additionally, outcomes suggesting improvements in social skills and prosocial behavior may also be associated with providing opportunities to safely role-play and model ER strategies during within-game activities and interactions. While there exist some mixed outcomes among qualitative research, video and app-based games may offer promising areas of research as psychoeducational tools to improve ER among youth.

### Mood Modification

This review also identified the potential use of video and app-based games as a means to alter or improve youth mood and engage in mood repair. Notably, 12 studies reported positive emotional and cognitive outcomes of engagement with these modalities, while also supporting the reduction in negative affect and beliefs. By engaging in games such as Bejeweled 2, Bookworm Adventures, Grow It!, ReGoal, and SquareMoves, outcomes suggested that youth are offered an opportunity to both disengage and reflect on their current emotions and mood, either heightening or decreasing their response in alignment with the process of attentional deployment. In doing so, these games may offer youth an opportunity to work through distressing thoughts and feelings while playing a fun game that allows them to take a break and reset their mood to feel ready to take on these feelings later at a lower arousal state. Dietvorst et al [55] also suggested that this was especially helpful among youth with higher risk profiles (eg, youth with a greater number of depressive symptoms and youth with parents who have psychiatric disorders). In particular, these video and app-based games may offer an immediate tool that has the potential to be used by youth alongside their parents to manage mood and mood repair during times of heightened stress. For example, O’Hara [75] highlighted in their study examining the utility of the JoyPop app within child welfare settings that the SquareMoves game may enhance self-focus to help promote reductions in parental stress and facilitate lowered risk of child maltreatment. However, given the mixed findings in the small number of studies where youth presented with greater mental health difficulties, balancing the use of video and app-based games to repair mood may need further investigation to understand this relationship and tolerance levels.

### Mental Health Symptom Management

Among the 20 studies investigated within this review, results suggested that video and app-based games were supportive in managing mental health disorder symptoms among youth in both nonclinical and clinical samples. For nonclinical youth experiencing anxiety symptoms, results appeared mixed. Regardless of sex or age group differences, some studies reported that video and app-based games have utility in reducing anxious symptoms, stress, and tension. However, other studies supported the notion that such modalities offered little reprieve from anxious symptoms and that these were only beneficial in the short term before traditional therapy sessions (eg, cognitive behavioral therapy), or even reported increases in state anxiety regardless of youth expectations of improvement from using the game [51]. In light of these mixed findings, anxiety and stress symptoms may be more transient experiences that may not serve as consistent targets for bolstering effective ER, and attempts at reduction or suppression may counterintuitively lead to heightened experiences [76]. Similarly, many of the included studies involved serious games with challenge or adventure elements that, while helping gamify learning for engagement, may neglect to emphasize consistent ER strategies. As such, it may be important for future studies to consider the balance between gamification and game-based learning when targeting anxiety and stress-related symptoms. This may include promoting the development of games that are designed with the intent of fostering learning, such as psychoeducation regarding effective ER strategies, as opposed to simple stress reduction through engaging gamification elements [77].

Among clinical youth, video and app-based games were used to address emotional difficulties in mental health disorders such as anxiety disorders, ASD, anger disorders, ADHD, and PTSD. However, similar to youth with subthreshold anxiety and stress symptoms, their utility in reducing clinical-level anxiety disorder symptomatology suggested mixed results. Additionally, among those with ASD, the examination by Wijnhoven et al [50] of ER outcomes appears to be due to a secondary diagnosis of an anxiety disorder as opposed to the autism spectrum diagnosis itself. Further, it appeared that using a VR-like game in the case of those with ADHD may also face limitations due to E1 and a need for a game to be more externally rewarding [66]. As such, video and app-based games may instead be better implemented as preliminary ER support and skills building, especially among youth experiencing anxiety symptoms, before engaging in services, supporting their potential utility as waitlist tools. In addition, studies suggested the potential use of such modalities in reducing aggression and disruptive behaviors among those with anger disorders. This may be due to the intentional approach to video and app-based games as intervention tools as opposed to other types of games (eg, action-packed and violent) that may promote more aggressive behavior among youth [78]. However, further research exploring the relationship between types of games as interventional tools to support ER among youth is warranted.

### Emotion Identification

While only captured within 4 studies, emotion identification emerged as a potential benefit of engaging with video and app-based games to encourage ER in youth. These results suggested that providing psychoeducation about emotions in such modalities may offer a more engaging way for youth to learn and express awareness of adaptive as opposed to maladaptive emotions. In doing so, such video and app-based games may serve as helpful tools in encouraging and bringing awareness to a youth’s emotional experiences in a stigma-free way, especially when such games may incorporate relatable avatars experiencing their own struggles with mental health [79]. This may be especially helpful among families with youth who have not benefited from traditional treatment and are looking for supportive, fun, and accessible tools to express or learn more about how they may be feeling. However, future research is warranted to explore this utility.

### Limitations and Strengths

This scoping review has several limitations as well as strengths. First, this review did not include quality assessments of the included studies themselves (eg, risk of bias). However, this review adhered to PRISMA-ScR standards via the inclusion of our study selection criteria and review of the available literature to provide as comprehensive a picture as possible of the current state of video and app-based games as interventions for ER. Second, this review did not capture video or app-based games used directly alongside treatments for the risk of losing the nuances offered by the games’ effects, specifically on ER outcomes. As such, future research would also benefit from investigating the use of video and app-based games when combined with treatment to also assess their clinical usefulness alongside services. Third, the initial pulling of studies for review was conducted by 3 independent reviewers. As such, this may have introduced a small degree of variance among the reviewers in terms of applying inclusion and exclusion criteria for the studies. However, given the systematic standards upheld for the research team, the impact of such variance is likely very low. Similarly, in order to capture psychological and mental-health-related constructs in relation to video and app-based game use, MEDLINE, Embase, and APA PsycInfo were selected for the scoping review. As such, other computer science and allied health databases may contribute additional papers and should be consulted in future reviews (eg, Web of Science, Scopus, IEEE Xplore, ACM Digital Library, and CINAHL). Lastly, the studies included tend to vary, in much the same way the literature does, by including studies of youth as young as 10 years and as old as 24 years. However, using this broad definition in the context of a scoping review allowed us to consider and be inclusive of the unique developmental needs of younger and older youth to promote ER.

### Conclusions

Overall, studies in this review suggest that video and app-based games may provide a promising modality for improving the ER abilities of youth, but require further investigation and rigorous study designs to address the heterogeneity of studies, youth samples, and mixed outcomes in the literature. As such, as additional research is published, considering a more systematic review or meta-analysis is warranted to look at the quality of emerging evidence. This scoping review provides a broad overview of the research to date and encourages suggestions for future directions regarding the state of ER technology for youth. As such, the review offers special consideration for the influence of app-based games alongside video games, given the growing field of mobile mental health apps supporting youth well-being. The noted ER outcomes offer promising areas of benefit that echo those of the process model of ER [7], while also suggesting ER considerations that may serve as the topics for future discussion and investigation as transdiagnostic treatment and support targets among youth. Notably, these findings may inform future research considering the role of video and app-based games for ER as viable stepped-care activities that youth may engage in to support themselves outside or before receiving formal treatment [80]. If supported, youth may benefit from using such games as early interventional tools to promote psychoeducation and transdiagnostic ER skills training that can better prime them for accessing supports. Alternatively, future research should explore whether these types of video and app-based games may better serve as adjunctive tools to facilitate in-treatment progress.

We hope this review can serve as an informative overview of the ER strategies and outcomes currently under investigation in emerging video and app-based games for youth. Given the mixed success of video and app-based games in the literature, this review has the potential to offer tangible support in the development of serious games that can build upon the methodological and design success of the featured video and app-based games. It will also be important for future research to delineate which video and app-based game features are more effective for supporting ER outcomes among younger youth (eg, those younger than 12 years) compared to the predominance of studies examining outcomes among the midadolescent and emerging adult age ranges. In doing so, we hope that future studies examining serious games can appropriately balance gamification popularized in casual games with skill-building ER targets for sustained engagement and wellness outcomes among the youth they are intended for.

## Data Availability

All data generated or analyzed during this study are included in this published paper and its supplementary information files.

## Appendix

Multimedia Appendix 1 PRISMA-ScR checklist.

Multimedia Appendix 2 Search strategy for databases.

Multimedia Appendix 3 Database of studies included in this scoping review.

## Abbreviations

ACE: adverse childhood experience
ADHD: attention-deficit/hyperactivity disorder
ASD: autism spectrum disorder
CBT: cognitive behavioral therapy
E1: levels of engagement
E2: health-related effectiveness
ER: emotion regulation
M: mechanisms of action
PRISMA: Preferred Reporting Items for Systematic Reviews and Meta-Analyses
PRISMA-ScR: Preferred Reporting Items for Systematic Reviews and Meta-Analyses extension for Scoping Reviews
PTSD: posttraumatic stress disorder
RCT: randomized controlled trial
T: target audience
TEME: target audience, engagement, mechanisms of action, and effectiveness
VR: virtual reality
