# Supplementary material for: Video and App-Based Games for Youth Emotion Regulation: Scoping Review
Source: JMIR Ment Health. 2026 Sep 9;13:e96935. doi: 10.2196/96935 (PMC13601871; doi:10.2196/96935)
Supplement: Supplementary file 2 [file mental_v13i1e96935_app2.docx]

**Search Strategy Across Databases**

MEDLINE

((video game or gaming) and (emotion or emotion regulation)).mp. [mp=title, book title, abstract, original title, name of substance word, subject heading word, floating sub-heading word, keyword heading word, organism supplementary concept word, protocol supplementary concept word, rare disease supplementary concept word, unique identifier, synonyms, population supplementary concept word, anatomy supplementary concept word]

((video game or gaming) and app).mp. [mp=title, book title, abstract, original title, name of substance word, subject heading word, floating sub-heading word, keyword heading word, organism supplementary concept word, protocol supplementary concept word, rare disease supplementary concept word, unique identifier, synonyms, population supplementary concept word, anatomy supplementary concept word]

(app and (emotion or emotion regulation)).mp. [mp=title, book title, abstract, original title, name of substance word, subject heading word, floating sub-heading word, keyword heading word, organism supplementary concept word, protocol supplementary concept word, rare disease supplementary concept word, unique identifier, synonyms, population supplementary concept word, anatomy supplementary concept word]

limit 1 to yr=”1902 - 2025"

Embase

('video game'/exp OR 'video game' OR 'gaming'/exp OR 'gaming') AND ('emotion'/exp OR 'emotion' OR 'emotion regulation'/exp OR 'emotion regulation') AND [<1966-2025]/py

('video game'/exp OR 'video game' OR 'gaming'/exp OR 'gaming') AND ('app'/exp OR 'app') AND [<1966-2025]/py

('app'/exp OR 'app') AND ('emotion'/exp OR 'emotion' OR 'emotion regulation'/exp OR 'emotion regulation') AND [<1966-2025]/py

APA PsychInfo

(video game) OR gaming AND emotion OR (emotion regulation)

((video game OR gaming) AND app)

(app AND (emotion OR emotion regulation))

Date: Before 31 December 2025
